# Clinicopsychological stratification of recovery outcomes in cerebral aneurysm patients following cluster analysis

**DOI:** 10.1093/pnasnexus/pgag157

**Published:** 2026-05-07

**Authors:** Sebastian R Reder, Jochen Hardt, Marc A Brockmann, Sven Rainer Kantelhardt, Axel Neulen, Verena Fassl, Katja Petrowski, Sabine Fischbeck

**Affiliations:** Department of Neuroradiology, University Medical Center, Johannes Gutenberg University Mainz, 55131 Mainz, Germany; Department of Medical Psychology and Medical Sociology, University Medical Center, Johannes Gutenberg University Mainz, 55128 Mainz, Germany; Department of Neuroradiology, University Medical Center, Johannes Gutenberg University Mainz, 55131 Mainz, Germany; Department of Neurosurgery, Vivantes Hospital Friedrichshain, 10249 Berlin, Germany; Department of Neurosurgery, University Medical Center, Johannes Gutenberg University Mainz, 55131 Mainz, Germany; Department of Neurosurgery, University Medical Center, Johannes Gutenberg University Mainz, 55131 Mainz, Germany; Department of Medical Psychology and Medical Sociology, University Medical Center, Johannes Gutenberg University Mainz, 55128 Mainz, Germany; Department of Medical Psychology and Medical Sociology, University Medical Center, Johannes Gutenberg University Mainz, 55128 Mainz, Germany

**Keywords:** brain aneurysm, quality of life, biopsychosocial, return to work, care planning

## Abstract

The aim of this present study was to investigate the multifactorial determinants of recovery outcomes in cerebral aneurysm patients by identifying subgroups based on clinical and psychological factors. A cross-sectional approach was used (*n* = 111), employing cluster analysis to group patients into minor deficits, moderate deficits, and severe deficits subgroups based on Short Form-36 Health Survey responses. Clinical outcomes were assessed using the modified Rankin scale (mRS), while psychological factors, such as anxiety, depression, and pain (including medication), were measured via self-reported questionnaires. AI- and MRI-based brain lesion volumes (LVs) were recorded (*n* = 82). Severe deficits demonstrated significantly worse clinical and psychological outcomes compared with minor deficits, including higher rates of aneurysm rupture, vasospasm, and larger LV. No significant differences in LV or work disability were found between moderate deficits and severe deficits. However, severe deficits showed significantly higher medication use for pain, sedation, and depression and more intense pain and psychological distress, including greater anxiety and perceived impairment due to the aneurysm diagnosis. This increased medication use was associated with poorer functional recovery and heightened psychological distress, suggesting that medication needs may reflect the severity of both physical and psychological impairment. The minor deficit group, being less affected by negative influences, demonstrated better overall outcomes. While LV and work disability did not differ between moderate deficits and severe deficits, significant disparities in medication use, mRS, perception of the cerebral aneurysm diagnosis, and psychological well-being were evident. These findings underscore the need for integrated care strategies addressing both physical and psychological factors to improve outcomes in cerebral aneurysm patients.

Significance Statement This study reveals a substantial divergence between expert-based functional outcome assessments (modified Rankin scale), structural brain damage (quantified by gliosis) and patients' self-perceived health status (Short Form-36 Health Survey scores) in individuals with brain aneurysms. Perceived illness is largely estimated through subjective parameters interpreted with the aid of expert-based instruments. However, a considerable mismatch exists between actual neurological impairment and the perceived severity of disease. These findings indicate that traditional expert-driven measures alone fail to fully capture patients' subjective health experience or the extent of underlying cerebral injury. Addressing these discrepancies requires an integrative evaluation approach that merges clinical expertise, imaging biomarkers, and patient-reported outcomes to better represent the true impact of aneurysmal disease.

## Introduction

Cerebral aneurysms present a significant health risk due to their potential to rupture, leading to hemorrhagic stroke, neurological damage, and death (1). This distinct form of hemorrhagic stroke constitutes ∼5% of all stroke cases in most Western nations (2), and mostly affects individuals in their fifth decade (3, 4). Advances in detection and treatment, particularly prerupture interventions such as surgical clipping or endovascular coiling, have improved outcomes (5, 6). However, less attention has been given to the psychological and psychosocial effects of living with an aneurysm, particularly in nonruptured cases.

Patients diagnosed with cerebral aneurysms often face emotional burdens due to the uncertainty surrounding their condition (7). The fear of rupture, along with concerns about cognitive or neurological decline, can significantly impact mental well-being. Many individuals with aneurysms experience anxiety, depression, and social withdrawal, which further diminish quality of life (QoL) (8, 9). These psychological issues are exacerbated by the physical symptoms, such as headaches, dizziness, and cognitive impairments, which may affect daily functioning (9, 10).

Early treatment of aneurysms generally leads to better outcomes, reducing the risk of rupture and improving long-term prognosis (5, 6). However, even with successful treatment, the psychological toll of living with a chronic condition remains. This study aims to assess both the physical and psychological impacts of cerebral aneurysms on patients.

Based on the population of Reder et al. (11), this research uses several data sources, including the Short Form-36 (SF-36) health survey, which evaluates health-related QoL (12). Advanced cerebral MRI (cMRI) data, analyzed using AI, will be used to assess gliotic and scar-related brain changes (or lesion volume [LV]) (13, 14). Additionally, digital subtraction angiography (DSA) will be employed to evaluate vasospasm (15), a complication that can worsen neurological deficits. Demographic factors such as age and gender will be considered, as well as the modified Rankin scale (mRS) to assess functional outcomes at discharge.

Psychological data are also incorporated through self-reported questionnaires, focusing on mental health challenges, such as anxiety and depression. By examining both physical and psychological outcomes, this study aims to provide a comprehensive understanding of how cerebral aneurysms affect patients' lives. By analyzing both the physical and psychological aspects of the disease, the study aims to identify whether physical deficits alone or a combination of physical and psychological factors contribute to the overall well-being of patients. It will also explore how factors like aneurysm treatment, rupture status, and complications, such as vasospasm, influence these outcomes. The findings from this study will contribute to more holistic care for cerebral aneurysm patients, emphasizing the importance of addressing both physical and psychological aspects in their management. This approach may lead to improved clinical outcomes and enhanced QoL for patients.

## Materials and methods

### Ethics approval and large language model usage statement

The study “AneuryCare” was conducted with approval from the Ethics Committee of the State Medical Association of Rhineland-Palatinate. The inclusion of aneurysm patients was authorized under the study number 837.366.17, 1120, and the application of patient-reported outcome measures (PROMs) in neurosurgical patients was authorized under the study number 837.097.15, 9865. This enabled initial telephone contact to be established to assess willingness to participate in the study. Informed consent was obtained during a subsequent phone call after participants had been informed about the study, and final confirmation was given thereafter. Additional study materials were then sent via email. Subsequently, additional study materials were sent via email. All procedures were performed in accordance with relevant ethical standards.

For grammatical correction, large language models were employed to enhance grammatical accuracy. These corrections were subsequently reviewed and deemed appropriate by all authors for final inclusion in the manuscript.

### Subject recruitment

The study included patients diagnosed with brain aneurysms who were discharged from the hospital between 2019 and 2022 (*n* = 207). One year after discharge, all patients were contacted by telephone and informed about the study; of the initial cohort, 96 patients declined participation, resulting in 111 patients who consented to take part. These 111 participants completed an online questionnaire. Recruitment was performed without prior panel affiliation, meaning that participants had not previously taken part in a comparable study and had not been informed in advance about the possibility of participation. After providing consent during the telephone call, participants were immediately enrolled and received a questionnaire link via email.

Of the 111 patients who participated in the survey, 82 had suitable cMRI and DSA data available for AI-based analysis. The cMRI data were sourced both from external radiological practices and from internal hospital facilities and were evaluated using AI models to assess aneurysm-related characteristics. The survey data were not weighted; all analyses were therefore based on the raw, unweighted responses.

The attrition rate was not systematically recorded, and as a result, no quantitative information on the proportion or characteristics of nonparticipants is available. Nonparticipation in the study may be partly explained by poorer clinical outcomes, for example, if patients with more severe disease courses or higher symptom burden were less likely or unable to participate. Such systematic differences between participants and nonparticipants could potentially bias the results, particularly if nonparticipants differ from respondents with respect to the variables under investigation.

### Online questionnaire content

The online questionnaire administered to participants consisted of several sections designed to capture demographic information, health-related QoL, anxiety, depression, and other psychosocial impact issues of a brain aneurysm diagnosis (11). Participants were first asked to provide basic demographic details, including age and gender. The SF-36 questionnaire was then used to assess their general health status (11, 16). The SF-36 scores are convertible into percentage values to facilitate comparisons with other population groups (11, 16). This transformation allows for a standardized assessment of health status, enabling a more meaningful comparison between the study cohort and broader reference populations. A cluster analysis was planned using the SF-36 questionnaire responses to stratify subgroups of participants. This approach aimed to identify distinct patterns in the subjective health status reported by participants. The resulting subgroups were then compared with objective (or expert-based) measurements to assess the alignment between self-reported health perceptions and clinically assessed outcomes.

To evaluate the personal stability and daily life impacts of their diagnosis, participants answered questions regarding how the aneurysm diagnosis affected their everyday activities. Additionally, work disability was assessed by asking participants to report the number of weeks per year they were unable to work due to health reasons (11).

Questions regarding relationship status and whether they had children provided insights into their social and family circumstances. Participants were also asked about their medication use, including any medications taken for pain, sleep aids, antidepressants, or sedatives. Medication use was assessed exclusively through a single question regarding current intake at the time of survey completion. Neither dosage information nor prior “ever used” history was systematically collected. Finally, the questionnaire inquired about the presence of psychiatric or psychological conditions, specifically focusing on depression, sleep disturbances, and anxiety.

#### Health-related QoL (SF-36)

The SF-36 (see Supplemental Material) is a well-researched outcome instrument for a self-reported measure of health-related QoL (11). It comprises 36 questions covering eight domains of health (11, 17): physical functioning, role—physical, bodily pain, general health, vitality, social functioning, role—emotional, and mental health (11). Two component scores, a physical health component score and a mental health component score, are derived from the eight subscales. A single item that assesses perceived change in health status over the past year was added. Higher scores on all subscales represent better health and functioning. Reliability scores of the SF-36 were Cronbach's *α* >0.85, reliability coefficient >0.75 for all dimensions except social functioning and for construct validity (distinguishing between groups with expected health differences) (18). The SF-36 questionnaire has still been used in aneurysm patient groups (19–22).

#### Physical domain

Physical functioning (PhyFunc) evaluates the impact of the health condition on activities like self-care, walking, climbing stairs, bending, lifting, and moderate or vigorous activities (11).

Physical role function (PhyRoFunc) assesses how the health condition affects work or daily activities, including reduced productivity, activity limitations, or difficulties in certain tasks (11).

General health (GenH) evaluates personal perceptions of health, encompassing current health status, future outlook, and ability to cope with illness and its aftermath (11).

Pain-related QoL (Pain) measures the intensity of pain and its interference with normal activities, both at home and elsewhere (11).

#### SF-36: mental domain

Social functioning (SoFunc) evaluates the extent to which physical health or emotional issues impair normal social activities (i.e. family life) (11).

Emotional role function (EmRoFunc) assesses the extent to which emotional problems affect work or other daily activities, including spending less time, accomplishing less, and not working as effortlessly as usual (11).

Vitality (Vit) assesses feelings of being energized and full of vigor versus tired and exhausted (11).

Psychological well-being (PsyWB) encompasses overall mental health, including depression, anxiety, emotional and behavioral control, and general positive mood (11).

#### Diagnosis-related (in-)stability

Each of the following statements was designed to reflect the perceived limitations in daily life due to the diagnosis of a brain aneurysm and was answered using a 5-point Likert scale with the options: “strongly disagree,” “disagree,” “neither agree nor disagree,” “agree,” and “strongly agree.”

- I am generally stable enough to live my life without worrying excessively about my aneurysm.
- I feel sensitive to even minor changes in my condition due to the aneurysm.
- I am constantly worried about whether and when my aneurysm will rupture.
- I have been psychologically unstable since the diagnosis of my aneurysm.
- I constantly worry about the possibility of my aneurysm enlarging.
- I am able to enjoy my life as I did before the aneurysm diagnosis.

#### Anxiety and depression (hospital anxiety and depression scale)

For the measurement of anxiety and depression, we used the hospital anxiety and depression scale (HADS) (23). It is a widely used self-report questionnaire in clinical and community settings. It consists of 14 items (seven related to anxiety and seven to depression). Each item is rated on a 4-point Likert scale, and the scores are summed to create separate anxiety and depression subscale scores. Cronbach's *α* for HADS-A varied from 0.68 to 0.93 (mean 0.83) and for HADS-D from 0.67 to 0.90 (mean 0.82). In most studies an optimal balance between sensitivity and specificity was achieved when caseness was defined by a score of 8 or above on both HADS-A and HADS-D (24).

### Digital subtraction angiography data and mRS at discharge

The findings from the interventional therapy, assessed through DSA, were derived from written reports obtained postprocedure. First, the rupture status was recorded (yes/no), indicating the presence or absence of aneurysm rupture at the time of the intervention. Additionally, the severity of vasospasm (none to severe) and the presence of residual aneurysm perfusion posttreatment (yes/no) were obtained.

The physical impairments (mRS) at hospital discharge were extracted from the documentation provided by the discharging department (neurosurgery).

### MRI and brain lesion volumetry

CMRI data were collected either following elective or acute therapy, with significant heterogeneity observed in the quality and consistency of the imaging. The majority of cMRIs were obtained from external facilities or local medical practices, while others were conducted internally at the institution. Both 1.5 and 3 T cMRI scanners were used for imaging acquisition. AI-assisted volumetric analysis of the brain LV was performed using the commercially available software “mdbrain” (Mediaire, Berlin, Germany). This analysis required T_2_-weighted sequences, which were only available in technically evaluable quality in 82 out of the 111 cases.

### Statistical analyses

The statistical analysis was performed using SPSS (version 29, IBM, Armonk, NY, United States). Cluster identification was carried out based on responses to the SF-36 questionnaire using Ward's method, with squared Euclidean distance and *Z*-standardization applied. For comparison of categorical variables, the Fisher's exact test was used, with a two-sided Monte Carlo significance test at a 99% CI. The Kruskal–Wallis test was employed to compare continuous variables across groups, with Bonferroni correction applied for multiple comparisons and two-sided adjusted significance levels. Additionally, the Kendall Tau-C test was applied to assess correlations between ordinal-scaled variables, with Monte Carlo significance testing at a 99% CI. All statistical tests were two-tailed, and a significance level of *P* < 0.05 was considered statistically significant unless otherwise specified.

## Results

The AneuryCare basic population included *n* = 83 women and *n* = 28 men (61.97 ± 12.29 vs. 61.25 ± 11.53 years).

### Cluster identification and stratification of results

A cluster analysis was conducted using Ward's method to identify subgroups within the study population based on SF-36 questionnaire responses. The resulting dendrogram identified three distinct clusters, saved for further analysis. The clusters were categorized as minor deficits, moderate deficits, and severe deficits, based on the participants' subjective health status.

### Basic population characteristics

The descriptive characteristics of the clustered population are summarized in Table 1. No significant differences were found between the clusters regarding age (*P* = 0.859) or gender distribution (*P* = 0.09). However, significant differences in LV (*P* = 0.003) were observed, with individuals in the severe deficits cluster showing notably larger LV compared with those in the minor deficit group.

**Table 1:** Associations between Various factors and cognitive deficits in patients with brain aneurysms stratified into the SF-36-questionnaire-based wardCluster minor, moderate, and severe deficits.

Factor	Minor deficits	Moderate deficits	Severe deficits	Total	*P*-value
Age (median, IQR)	64.00 (13.00)	63.50 (16.00)	64.50 (13.50)	136	0.859
Lesion volume (median, IQR)	2.75 (3.57)	5.35 (11.14)	6.11 (6.22)		**0.003**
Gender					**0.09**
Male	20	10	13	43	
Female	28	37	28	93	
Rupture status					**0.002**
Nonruptured	11	20	12	43	
Ruptured	0	4	8	12	
Residual aneurysm perfusion					0.221
No	4	8	12	24	
Yes	7	16	8	31	
Relationship status					0.966
No	10	11	10	31	
Yes	35	35	30	100	
Children					0.420
None	9	18	10	37	
Yes	38	29	30	97	
Pain medication use					**<0.001**
Daily	1	4	6	11	
Several times per week	3	2	8	13	
Less frequently	16	17	15	48	
Never	26	21	11	58	
Sleeping medication use					**0.05**
Daily	0	2	2	4	
Several times per week	1	2	1	4	
Less frequently	2	3	5	10	
Never	42	37	31	110	
Sleep disturbances					**0.033**
No	44	40	32	116	
Yes	3	6	9	18	
Depression medication use					**0.007**
Daily	2	2	6	10	
Several times per week	0	1	1	2	
Less frequently	0	1	3	4	
Never	44	40	29	113	
Sedative medication use					**0.012**
Daily	0	1	2	3	
Several times per week	0	0	1	1	
Less frequently	1	3	4	8	
Never	44	40	31	115	
Anxiety disorder					0.869
No	21	25	26	72	
Yes	1	3	2	6	

This table presents the associations between various demographic, health, and psychological factors and cognitive deficits (minor, moderate, and severe) in patients diagnosed with a brain aneurysm.

Regarding rupture status, a significant association appeared (*P* = 0.002). The severe deficit group had a higher proportion of ruptured aneurysms, with eight out of 20 patients experiencing rupture, while no ruptured cases were reported in the minor deficit group. No significant differences were observed in residual aneurysm perfusion (*P* = 0.221).

Sleep disturbances occurred more frequently in the moderate (*P* = 0.033) and severe deficit groups, with nine out of 41 participants in the severe deficit group reporting sleep issues. Anxiety disorder showed no significant association with the clusters (*P* = 0.869), as the prevalence remained low across all groups.

Relationship status and presence of children as potential protective factors did not show significant differences between clusters, with *P*-values of 0.966 and 0.420, respectively.

Medication use patterns varied across clusters. Medication use data (Table 1) reflect current intake at survey time rather than lifetime exposure or dosage. Pain medication use was significantly higher in the severe deficit group (*P* < 0.001), with six individuals using pain medication daily, compared with only one in the minor deficit group. Sleeping medication use showed a trend toward significance (*P* = 0.05), with the severe deficit group reporting more frequent use. Depression medication use was significantly higher in the severe deficit group (*P* = 0.007), with six participants using antidepressants daily. Sedative medication use differed significantly across clusters (*P* = 0.012), with the severe deficit group reporting higher usage.

### SF-36 results

**Figure 1:**
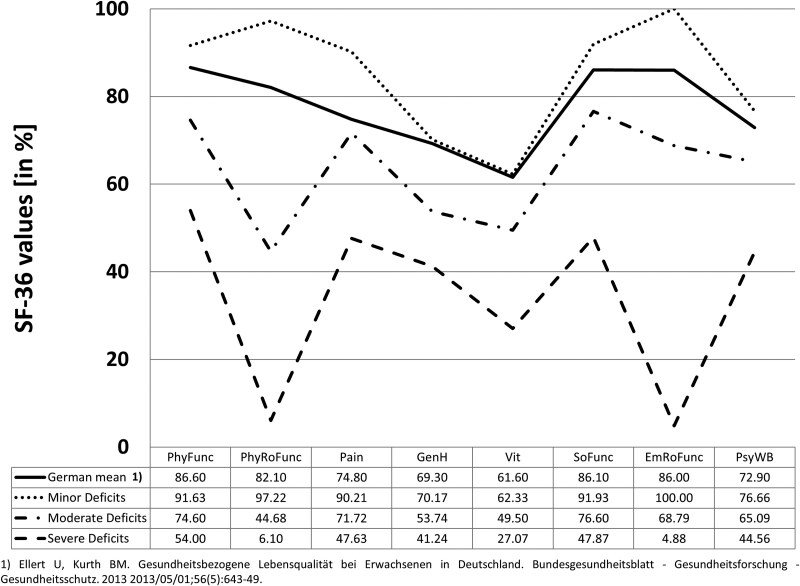
SF-36-based clusters compared with the German normal population. This figure illustrates the comparison of three clusters, minor deficits, moderate deficits, and severe deficits, based on SF-36 questionnaire responses, with the German normal population, obtained from Ellert and Kurth (25). The clusters were extracted through cluster analysis of the SF-36 data and compared across several health-related quality of life dimensions: physical functioning (PhyFunc), physical role function (PhyRoFunc), general health (GenH), pain-related quality of life (Pain), social functioning (SoFunc), emotional role function (EmRoFunc), vitality (Vit), and psychological well-being (PsyWB). Higher values on these scales reflect better quality of life as perceived by individuals (in %). The figure highlights how the three patient subgroups (minor, moderate, and severe deficits) differ from the normal population and from each other in terms of their self-reported health outcomes. Notably, the severe deficit group shows significantly lower scores across most domains, indicating a poorer quality of life compared with the other two groups and the general population.

The mean scores of the SF-36 subscales were compared with the overall German population (25) and the three identified AneuryCare subgroups (minor deficits, moderate deficits, and severe deficits; see Fig. 1). Higher SF-36 scores indicate better self-reported perceived health, while lower scores reflect poorer health.

#### Physical domains

For the physical functioning (PhyFunc) subscale, the German population (25) had a mean score of 86.60. Participants in the minor deficit group exhibited a higher score (91.63), while those in the moderate and severe deficit groups had lower scores, 74.60 and 54.00, respectively. A similar trend was observed in physical role functioning (PhyRoFunc), with the minor deficit group scoring the highest (97.22), followed by the German population (25) (82.10), and the moderate and severe deficit groups at 44.68 and 6.10, respectively. In terms of pain (Pain), the minor deficit group again scored the highest (90.21), significantly higher than both the moderate deficit group (71.72) and the severe deficit group (47.63), with the German population (25) mean at 74.80. General health (GenH) showed lower scores in all subgroups compared with the German population (25) mean (69.30), with the minor deficit group scoring 70.17, the moderate deficit group at 53.74, and the severe deficit group at 41.24.

#### Mental domains

For vitality (Vit), the minor deficit group had a slightly higher score (62.33) than the German population (61.60) (25), while the moderate and severe deficit groups scored notably lower, with values of 49.50 and 27.07, respectively. The social functioning (SoFunc) scores demonstrated a similar trend, with the minor deficit group reporting a significantly higher score (91.93) than both the moderate (76.60) and severe deficit groups (47.87), in comparison to the German population mean of 86.10 (25). Emotional role functioning (EmRoFunc) showed a substantial disparity, with the minor deficit group scoring the highest (100.00), followed by the German population (86.00) (25). The moderate deficit group scored 68.79, and the severe deficit group had a significantly lower score of 4.88. Lastly, the psychological well-being (PsyWB) subscale revealed a similar distribution, with the minor deficit group scoring the highest (76.66), the German population at 72.90 (25), and the moderate (65.09) and severe deficit groups (44.56) showing progressively lower scores.

**Figure 2:**
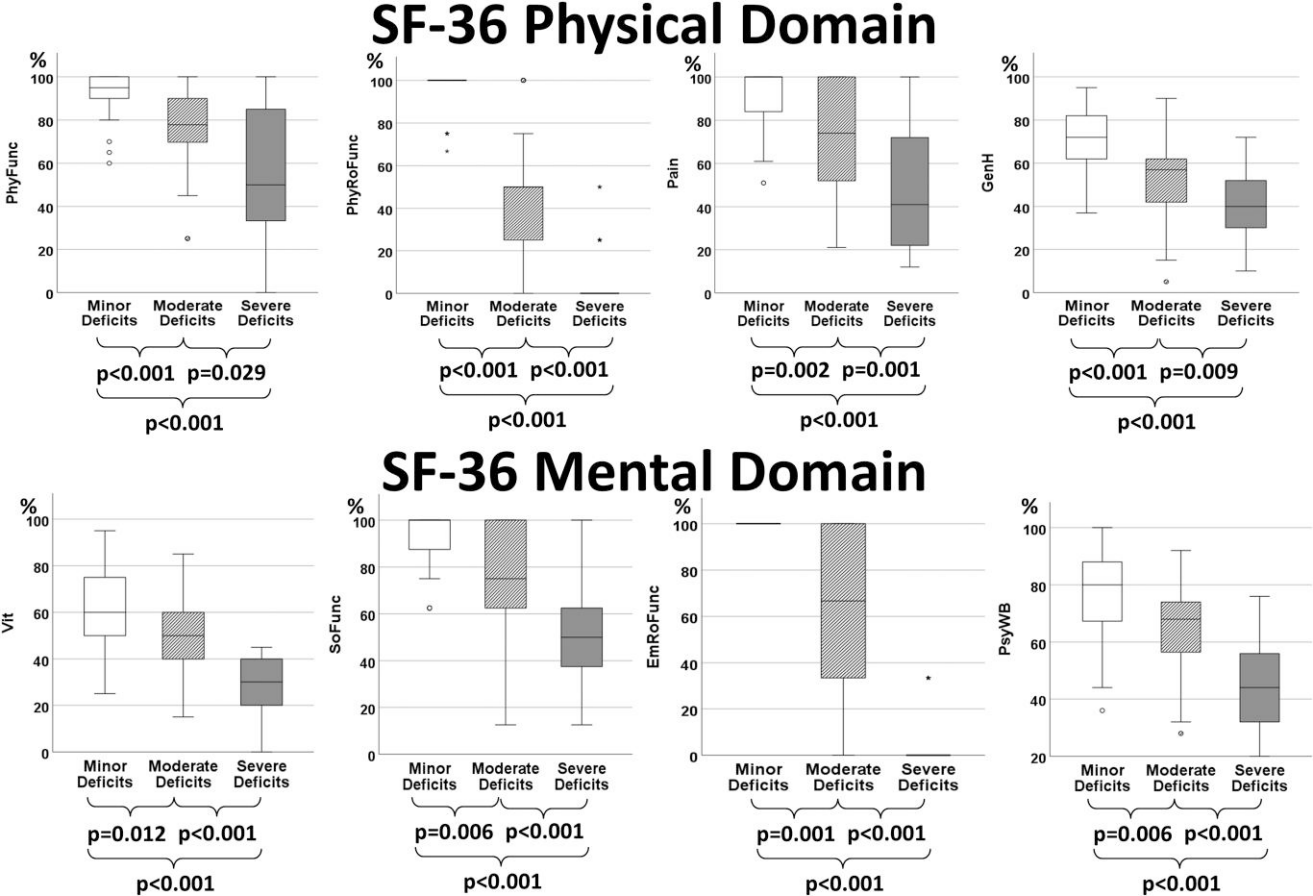
Boxplot analysis of SF-36 scores across the three clusters. This figure presents boxplots comparing the individual SF-36 scores between the three clusters: minor deficits, moderate deficits, and severe deficits. The analysis includes scores from both the physical and mental domains of the SF-36. The physical domain consists of physical functioning (PhyFunc), physical role function (PhyRoFunc), general health (GenH), and pain-related quality of life (Pain), while the mental domain includes social functioning (SoFunc), emotional role function (EmRoFunc), vitality (Vit), and psychological well-being (PsyWB). The boxplots demonstrate distinct differences across all variables between the three clusters, indicating that the severity of physical and psychological deficits significantly impacts patients' reported health outcomes. Higher values on these scales reflect better quality of life as perceived by individuals (in %). The severe deficit group shows consistently lower scores in both domains, suggesting a greater impairment in QoL.

In the analysis of the medians of the stratified SF-36 scores (Kruskal–Wallis test), all results were found to be significantly different between the three subgroups (Fig. 2). These results highlight the significant variation in health-related QoL across the identified subgroups, with participants in the severe deficit group showing the greatest impairments across all subscales.

### Diagnosis-related (in-)stability

The analysis of psychological stability among the three subgroups revealed significant differences in the responses to several key items (Table 2).

**Table 2:** Psychological stability and concerns regarding the diagnosis brain aneurysm.

Psychological stability	Minor deficits	Moderate deficits	Severe deficits	*P*-value
**I am generally stable enough to live my life without worrying excessively about my aneurysm**				**<0.001**
Strongly disagree	2	2	3	
Disagree	1	6	10	
Neither agree nor disagree	3	4	10	
Agree	18	20	13	
Strongly agree	24	14	5	
**I feel sensitive to even minor changes in my condition due to the aneurysm**				**0.006**
Strongly disagree	17	14	7	
Disagree	10	3	6	
Neither agree nor disagree	5	4	7	
Agree	13	22	10	
Strongly agree	3	3	11	
**I am constantly worried about whether and when my aneurysm will rupture**				**0.037**
Strongly disagree	17	13	12	
Disagree	21	9	8	
Neither agree nor disagree	6	10	13	
Agree	3	11	4	
Strongly agree	1	3	4	
**I have been psychologically unstable since the diagnosis of my aneurysm**				**<0.001**
Strongly disagree	27	24	7	
Disagree	15	14	12	
Neither agree nor disagree	3	5	10	
Agree	1	1	7	
Strongly agree	2	1	5	
**I constantly worry about the possibility of my aneurysm enlarging**				**<0.001**
Strongly disagree	22	16	8	
Disagree	12	8	7	
Neither agree nor disagree	9	12	13	
Agree	5	7	7	
Strongly agree	0	3	5	
**I am able to enjoy my life as I did before the aneurysm diagnosis**				**<0.001**
Strongly disagree	5	2	10	
Disagree	3	5	7	
Neither agree nor disagree	6	12	15	
Agree	21	18	7	
Strongly agree	13	9	2	

The psychological stability measures include various aspects of life satisfaction and anxiety related to the aneurysm. The self-assessed deficits in cognitive function were categorized into minor, moderate, and severe based on the SF-36 questionnaire.

Regarding the statement “I am generally stable enough to live my life without worrying excessively about my aneurysm,” the results were significantly different across the subgroups (*P* < 0.001). The minor deficit group reported the highest level of agreement, with 24 individuals strongly agreeing, compared with 14 in the moderate deficit group and five in the severe deficit group.

For the statement “I feel sensitive to even minor changes in my condition due to the aneurysm,” significant differences were also observed (*P* = 0.006). The minor deficit group exhibited lower sensitivity, with 17 individuals strongly disagreeing, while the severe deficit group reported higher sensitivity, with 11 individuals strongly agreeing.

The statement “I am constantly worried about whether and when my aneurysm will rupture” showed a significant difference across the subgroups (*P* = 0.037). While the minor deficit group demonstrated a lower level of concern, with 17 individuals strongly disagreeing, the severe deficit group had a higher proportion of individuals strongly agreeing (four individuals).

In response to “I have been psychologically unstable since the diagnosis of my aneurysm,” significant differences were found (*P* < 0.001). The minor deficit group exhibited the lowest levels of psychological instability, with 27 individuals strongly disagreeing. In contrast, the severe deficit group had the highest levels of instability, with five individuals strongly agreeing.

The statement “I constantly worry about the possibility of my aneurysm enlarging” showed a similar pattern (*P* < 0.001). The minor deficit group had the highest number of individuals strongly disagreeing (22 individuals), while the severe deficit group had the highest proportion of individuals strongly agreeing (five individuals).

Lastly, in response to the statement “I am able to enjoy my life as I did before the aneurysm diagnosis,” significant differences were observed (*P* < 0.001). The minor deficit group showed the highest levels of enjoyment, with 13 individuals strongly agreeing, while the severe deficit group had only two individuals strongly agreeing.

These findings indicate that psychological stability significantly varies between the three subgroups, with the severe deficit group reporting higher levels of worry, instability, and concerns about aneurysm-related changes. Conversely, the minor deficit group reported greater psychological stability and a better ability to enjoy life postdiagnosis.

### Investigation of the cause-effect principle from acute phase to 1 year postacute

**Figure 3:**
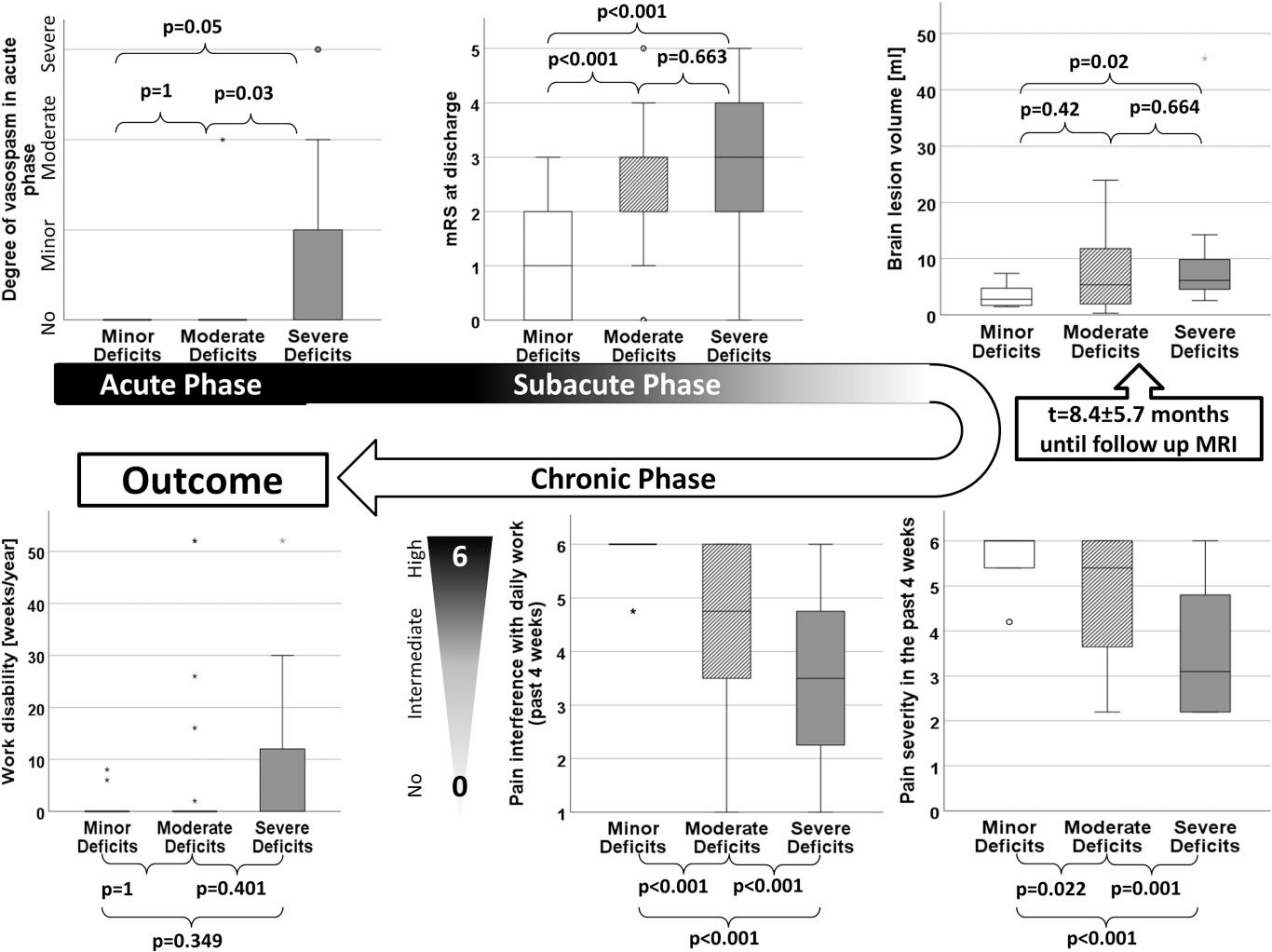
Potential causes of subjective health perception across disease stages. This figure outlines the potential causes of subjective health perception in patients with cerebral aneurysms, explored through multiple stages of the disease, ranging from the acute phase to 1 year postacute. The analysis begins with the acuteness of the diagnosis, categorized by rupture status and vasospasm presence. This is followed by the subacute phase at discharge, where physical impairment was assessed using the mRS. Residual brain lesions were evaluated ∼8.4 ± 5.7 months after the acute phase using AI-based brain lesion volumetry. The chronic phase was examined through the analysis of pain levels reported in the 4 weeks prior to the survey and self-reported work disability within the past year. Key findings include a higher frequency of ruptures and more severe vasospasms in the severe deficit group compared with the minor deficit group (*P* = 0.03 and *P* = 0.05, respectively). At discharge, the severe deficit group also exhibited significantly worse functional outcomes, as measured by the mRS, in comparison to the minor deficit group (*P* < 0.001), with no significant differences between the moderate and severe deficit groups (*P* = 0.663). cMRI findings indicated significant differences in brain LVs, with the severe deficit group showing larger lesions than the minor deficit group (*P* = 0.02), but no significant differences were found between the moderate and severe deficit groups (*P* = 0.664). Additionally, the severe deficit group reported significantly higher levels of pain that had a greater impact on their daily life compared with the minor and moderate deficit groups (pain in minor < moderate < severe; each *P* < 0.05). Interestingly, no significant differences were found in the duration of work disability between the groups.

The potential causes of the subjective health perception were investigated through various stages, ranging from the acute phase to 1 year postacute (Fig. 3). The analysis focused on the acuteness of the diagnosis, categorized by rupture status and the presence of vasospasm, and proceeded through the subacute phase at discharge, where physical impairment was assessed using the mRS. Further, residual brain lesions 8.4 ± 5.7 months following the acute phase were evaluated through cMRI-based brain lesion volumetry, with findings presented in the Basic population characteristics section and Table 1. The chronic phase was explored by analyzing pain reported in the 4 weeks preceding the survey and self-reported work disability within the past year.

The severe deficit group exhibited a higher frequency of ruptures and more severe vasospasms compared with the minor deficit group (*P* = 0.03 and *P* = 0.05, respectively). At discharge, the severe deficit group also demonstrated a significantly worse functional status, as assessed by the mRS, in comparison to the minor deficit group (*P* < 0.001), with no significant differences observed between the moderate and severe deficit groups (*P* = 0.663). CMRI findings at follow-up revealed significant differences in brain LV, with the severe deficit group showing a higher LV compared with the minor deficit group (*P* = 0.02). However, no significant differences in LV were found between the moderate and severe deficit groups (*P* = 0.664). The severe deficit group also reported significantly higher levels of pain, which they indicated had a greater impact on their daily life, compared with both the minor and moderate deficit groups (pain in minor < moderate < severe; each *P* < 0.05). Interestingly, no significant differences were observed in the duration of work disability between the groups.

## Discussion

This study aimed to investigate the subjective health experiences of brain aneurysm patients by identifying distinct subgroups based on SF-36 questionnaire responses, followed by a comparison with objective clinical measures across different stages of the disease. The analysis revealed three clusters: minor deficits, moderate deficits, and severe deficits, which were further examined in terms of demographic, clinical, psychological, and health-related outcomes.

The hypothesis guiding this study posited that both physical and psychological factors contributed significantly to the overall well-being of patients with cerebral aneurysms. It was anticipated that a combination of physical deficits and psychological challenges would have a greater impact on patient QoL than physical deficits alone. By analyzing both physical and psychological outcomes, the study aimed to provide a comprehensive understanding of how these factors influenced recovery and health-related QoL. The findings from this study confirmed that the severity of both physical deficits and psychological distress contributed significantly to the overall health status of patients, aligning with this hypothesis.

Cluster analysis revealed that patients in the severe deficit group had more aneurysm ruptures and severe vasospasms, associated with larger LV compared with the minor deficit group and worse functional outcomes as measured by the mRS at discharge (26). These findings suggested that the severity of the aneurysm treatment and its complications (rupture and vasospasm) affected functional recovery and overall health status (27, 28). Larger brain lesions, typically resulting from more extensive vasospasms, were correlated with poorer subjective health outcomes (26). These results aligned with previous research that highlighted the relationship between greater brain injury and worse recovery trajectories after aneurysm rupture (15, 26–30). However, no significant differences in LV were found between the moderate and severe deficit groups. This observation suggested that while lesion size was important, other factors such as lesion location (31, 32) might have also contributed to the severity of clinical outcomes.

The study's female predominance (75%) aligns with established epidemiological patterns, where women exhibit both higher prevalence and rupture risk of intracranial aneurysms (33). Beyond reflecting our neurovascular center's source population, this disparity likely stems from multifaceted sex-dichotomous mechanisms. Epigenetic modifications demonstrate pronounced gender effects: vestigial-like family member 3 (VGLL3) promoter hypermethylation correlates positively with female-specific lipoprotein(a) levels and negatively with age and apolipoproteine E (APOE) expression, potentially promoting aneurysm pathogenesis via VGLL3/transcriptional enhanced associate domain family members (TEAD)/yes-associated protein (YAP) pathway dysregulation (34). Similarly, nitric oxide synthase 1 adaptor protein (NOS1AP) promoter DNA methylation exerts sex-specific influences on aneurysm formation, highlighting differential endothelial nitric oxide signaling vulnerabilities (35). Connective tissue disorders further amplify this risk. Polycystic kidney disease patients show elevated aneurysm prevalence, with female predominance linked to extracellular matrix remodeling defects (36). Classic risk factor analyses confirm hormonal influences—particularly estrogen fluctuations—and hemodynamic stressors as key contributors to earlier rupture peaks in women (33, 37). Collectively, these genetic, epigenetic, and structural factors underscore why female patients dominated our cohort while also suggesting sex-stratified approaches for future biopsychosocial outcome research (33).

Medication use patterns varied across the groups. The severe deficit group exhibited higher use of pain, sleep, and depression medications, underscored by several publications (38–40). These results underscored the ongoing burden of symptoms in this group, suggesting that pharmacological interventions alone may not have fully addressed the complex needs of these patients (39). A more comprehensive care approach, incorporating both pharmacological and psychological support, appeared essential for improving patient outcomes.

Psychologically, patients in the severe deficit group reported significantly higher levels of anxiety, greater concerns about rupture, and more psychological instability (39). Additionally, they experienced persistent pain, which had a greater impact on daily life (39, 41). Despite these differences in pain and psychological well-being, no significant differences in the duration of work disability were observed between the groups (39).

While psychological stability across subgroups presented striking differences, certain potential protective factors were explored but did not explain the differences between the moderate and severe deficit groups. Although the perception of threat posed by diseases can be influenced by gender-specific factors (42), this variable does not appear to play a significant role in this context. Furthermore, factors such as the presence of children, a stable relationship, and residual aneurysm perfusion were not associated with the disparities observed between these groups. Despite the higher frequency of vasospasm in the severe deficit group, no significant differences in LV were found between the moderate and severe groups. This observation points to the possibility that additional factors beyond lesion size, such as lesion location or patterns of brain injury, could account for these differences (31). In addition, the minor deficit group unexpectedly scored higher on SF-36 domains than normative data, despite reporting the highest pain levels with the lowest medication use. While posttraumatic health reappraisal or coping resilience may explain this discrepancy (“responsive shift”), definitive mechanisms require longitudinal validation (11, 43, 44). Factors such as coping mechanisms, social support, or work-related accommodations might have mitigated the impact of these symptoms on employment.

Further investigation into potential protective factors is essential to better understand these variations. For instance, lesion patterns in the brain, particularly in areas such as the frontal, temporal, or thalamic regions, might contribute to the severity of symptoms (45–47). These areas are known to play key roles in emotional regulation and pain processing, and damage to these regions may lead to poorer self-assessment of one's condition or increased pain sensitivity (45–48). This could help explain the significantly higher mRS scores at discharge in the severe deficit group, indicating greater functional impairment compared with other groups (31, 49). Additionally, lesions in brain regions responsible for cognitive and emotional processing might exacerbate the psychological distress reported by the severe deficit group, potentially complicating their recovery (31). Another factor that may play a significant role in the severity of symptoms is individual susceptibility to stress, particularly chronic stress, and its impact on pain disorders and depression (48, 50–52). Long-term stress has been shown to influence the body's immune response, including cytokine production, and this may, in turn, interact with neurological processes (51–53). Chronic stress can lead to an imbalance in cytokine levels, which could affect brain function and increase vulnerability to pain and depression (51–53). This physiological mechanism may help explain the heightened pain sensitivity and psychological distress observed in the severe deficit group. Further research into how stress, cytokine regulation, and individual stress susceptibility interact could provide valuable insights into improving both the physical and psychological outcomes for cerebral aneurysm patients.

Several limitations should be acknowledged. First, the relatively small cohort size limits the statistical power and generalizability of the findings. Given the specific requirements for comprehensive clinical, psychological, and imaging data, recruitment was constrained to patients who had complete datasets of sufficient technical quality. Accordingly, the present results should be regarded as exploratory and hypothesis generating rather than confirmatory. The study primarily utilized cross-sectional data, limiting the ability to establish causal relationships between clinical factors, psychological outcomes, and health-related QoL. Longitudinal studies are needed to better understand how these factors evolve over time and influence recovery trajectories. Larger sample sizes would provide more robust data and enhance the statistical power of the study. Additionally, psychological outcomes, including anxiety, depression, and pain, were measured using self-reported questionnaires. These subjective measures may be prone to reporting biases, such as over- or under-reporting of symptoms. Objective measures of psychological distress or clinician-rated assessments could complement these findings. Furthermore, several potential confounders, such as comorbidities, socioeconomic status, and lifestyle factors, were not fully accounted for in the analysis. A potential selection bias cannot be excluded. During telephone recruitment, patients were only asked for consent to participate, but reasons for declining were not systematically documented. Consequently, no detailed information is available on why 96 patients (46%) declined participation. It is conceivable that individuals with severe neurological disability, death, comorbidities, or emotional burden were underrepresented, leading to a potentially healthier or more functional study sample. This limitation may affect the generalizability of our findings. These factors could influence both physical and psychological outcomes and should be considered in future studies to control for their potential impact. Although cMRI findings were included in the analysis, various factors could influence the interpretation of LV. These factors include the timing of the cMRI relative to the acute event, variations in imaging protocols, and the subjective nature of LV measurements. Lastly, while the study examined the impact of vasospasm and rupture status on clinical outcomes, it did not fully explore the effects of treatment protocols, such as surgical versus conservative management, on recovery.

## Conclusion

This study provides several insights into health-related outcomes after cerebral aneurysm by identifying distinct patient subgroups that differ in both physical and psychological domains. Our findings demonstrate that self-reported disease severity represents a valid and clinically meaningful dimension of biopsychosocial impairment in this population. Because self-perceptions remain the only directly accessible indicators of disease burden, they were deliberately used as the basis for clustering and subsequent multimodal analysis. The identification of three subgroups derived from SF-36 responses allowed a more nuanced understanding of how clinical and psychological factors interact to shape long-term recovery.

Importantly, the study revealed both expected and unexpected relationships between self-reported and objective findings. The results highlight that aneurysm severity—particularly rupture and vasospasm—strongly influences functional and emotional outcomes. Patients in the severe deficit group showed poorer results across both clinical and psychological measures, with greater levels of anxiety, pain, and depression contributing substantially to overall health impairment. This group also required more frequent use of antidepressants, sedatives, and analgesics to maintain work capacity, underscoring the need for integrated care strategies that address both physical and psychological challenges. Conversely, subjective disease appraisal did not merely mirror measurable pathology but added valuable complementary information explaining discrepancies between brain damage and perceived suffering. Interestingly, patients classified as having moderate deficits based on self-report exhibited a higher lesion load on MRI compared with those in the severe deficit group, while participants in the minor deficit group reported the highest pain burden in daily life despite the lowest medication intake. Regarding work incapacity, there was an overall trend toward longer absence in the severe deficit group but without statistical significance, likely due to the single-center design.

This multidimensional perspective advances a more holistic understanding of postaneurysm outcomes and lays the groundwork for future multicenter studies exploring potential biological and psychosocial mediators, including the role of chronic low-grade inflammation.

Finally, our observations suggest that lesion patterns in brain regions involved in emotional regulation and pain processing may contribute to symptom severity, as damage in these areas could exacerbate both functional deficits and psychological distress. Moreover, individual stress vulnerability and its interaction with immune mechanisms—such as cytokine activity—may further influence the emergence of pain and depression, highlighting the importance of continued research into these neurobiological pathways.

## Supplementary Material

pgag157_Supplementary_Data

## Data Availability

Data are available at the data holding institute. Inquiries must be sent to the director. Each request should be based on a scientific hypothesis and have been reviewed by a (local) ethical committee. Any request must be made in writing. Data will be saved for 10 years after publication (according to the Good Clinical Practice guidelines).
