# Supplementary material for: Clinicopsychological stratification of recovery outcomes in cerebral aneurysm patients following cluster analysis
Source: PNAS Nexus. 2026 May 7;5(5):pgag157. doi: 10.1093/pnasnexus/pgag157 (PMC13195298; doi:10.1093/pnasnexus/pgag157)
Supplement: pgag157_Supplementary_Data [file pgag157_supplementary_data.docx]

**Measurement of Quality of Life (SF‑36)**

In this questionnaire you are asked to rate your health status. The form allows us to track how you feel and how you manage in everyday life over time.

Please answer each of the following questions by selecting, among the options, the number that best applies to you.

**General Health Status**

a) How would you describe your general health status? *(Please mark only one number.)*

O  excellent
O  very good
O  good
O  less good
O  poor

b) Compared with last year, how would you describe your current health status? *(Please mark only one number.)*

O  much better now than a year ago
O  somewhat better now than a year ago
O  about the same as a year ago
O  somewhat worse now than a year ago
O  much worse now than a year ago

**ACTIVITIES table**

Below are some activities that you might perform on an ordinary day. Are you limited in these activities by your current health status? If so, how limited? *(Please mark only one number in each row.)*

| **ACTIVITIES** | **Yes, strongly limited** | **Yes, somewhat limited** | **Not limited at all** |
| --- | --- | --- | --- |
| a. strenuous activities, e.g., running quickly, lifting heavy objects, taking part in strenuous sport |  |  |  |
| b. moderate activities, e.g., moving a table, vacuum cleaning, bowling, playing golf |  |  |  |
| c. lifting or carrying shopping bags |  |  |  |
| d. climbing several flights of stairs |  |  |  |
| e. climbing one flight of stairs |  |  |  |
| f. bending, kneeling, stooping |  |  |  |
| g. walking more than 1 kilometer |  |  |  |
| h. walking several street blocks |  |  |  |
| i. walking one street block |  |  |  |
| j. bathing or dressing yourself |  |  |  |

**DIFFICULTIES (physical) table**

In the past 4 weeks, have you had any difficulties at work or in other daily activities at home because of your physical health? *(Please mark only one number in each row.)*

| **DIFFICULTIES** | **Yes** | **No** |
| --- | --- | --- |
| a. I could not be active as long as usual |  |  |
| b. I got less done than I wanted |  |  |
| c. I could only do certain things |  |  |
| d. I had difficulties performing tasks (e.g., I had to exert myself especially) |  |  |
| a. I could not be active as long as usual |  |  |

**DIFFICULTIES (mental) table**

In the past 4 weeks, have you had any difficulties at work or in other daily activities at home because of emotional or psychological problems (for example, because you felt depressed or anxious)? (*Please mark only one number in each row.)*

| **DIFFICULTIES** | **Yes** | **No** |
| --- | --- | --- |
| a. I could not be active as long as usual |  |  |
| b. I got less done than I wanted |  |  |
| c. I could not work as carefully as usual |  |  |

**How much have your physical health or emotional problems in the past 4 weeks affected your normal contacts with family members, friends, neighbors, or acquaintances?**

*(Please mark only one number)*

O not at all

O  somewhat

O  moderately

O  quite a bit

O  very much

**How strong were your pains in the past 4 weeks?** *(Please mark only one number)*

O  I had no pain

O  very mild

O  mild

O  moderate

O  strong

O  very strong

**To what extent did pain restrict you in carrying out your everyday activities at home and at work in the past 4 weeks?** *(Please mark only one number)*

O  not at all

O  somewhat

O  moderately

O  quite a bit

O  very much

**WELL‑BEING table (frequency)**

These questions concern how you feel and how you have been feeling in the past 4 weeks. (Please mark, in each row, the number that best describes your well‑being.) How often were you in the past 4 weeks… *(Please mark only one number in each row.)*

| **WELL‑BEING** | **Always** | **Mostly** | **Quite often** | **Sometimes** | **Rarely** | **Never** |
| --- | --- | --- | --- | --- | --- | --- |
| a. full of energy / peppy |  |  |  |  |  |  |
| b. very nervous |  |  |  |  |  |  |
| c. so depressed that nothing could cheer you up |  |  |  |  |  |  |
| d. calm and relaxed |  |  |  |  |  |  |
| e. full of energy |  |  |  |  |  |  |
| f. discouraged and sad |  |  |  |  |  |  |
| g. exhausted |  |  |  |  |  |  |
| h. happy |  |  |  |  |  |  |
| i. tired |  |  |  |  |  |  |

**How often have your physical health or emotional problems in the past 4 weeks affected your contacts with other people (visits to friends, relatives, etc.)?** *(Please mark only one number.)*

O  always

O  most of the time

O  sometimes

O  rarely

O  never

**To what extent does each of the following statements apply to you?** *(Please mark only one number in each row.)*

|  | **Completely true** | **Mostly true** | **Don't know** | **Mostly not true** | **Not true at all** |
| --- | --- | --- | --- | --- | --- |
| a. I seem to get sick more easily than others |  |  |  |  |  |
| b. I am just as healthy as the other people I know |  |  |  |  |  |
| c. I expect that my health will decline |  |  |  |  |  |
| d. I enjoy excellent health |  |  |  |  |  |

**How would you describe your current health status?** *(Please mark only one number.)*

O  very good
O  good
O  fair
O  poor
O  very poor

**WELL‑BEING (short yes‑no statements) table**

In the following you will find a series of statements. Please mark (X) in each row whether this applies to you or not.

|  | **Yes** | **No** |
| --- | --- | --- |
| I am constantly tired. |  |  |
| I have pain at night. |  |  |
| I feel depressed. |  |  |
| I have unbearable pain. |  |  |
| I take tablets to be able to sleep. |  |  |
| I have forgotten what it feels like to experience joy. |  |  |
| I feel irritable. |  |  |
| I find it painful to change my body position. |  |  |
| I feel lonely. |  |  |
| I can only move within the house. |  |  |
| It is difficult for me to bend down. |  |  |

**CONTINUATION “WELL‑BEING” table**

|  | **Yes** | **No** |
| --- | --- | --- |
| Everything exhausts me. |  |  |
| I wake up very early in the morning. |  |  |
| I cannot walk at all. |  |  |
| It is difficult for me to make contact with other people. |  |  |
| The days drag on. |  |  |
| I have difficulties going up and down stairs. |  |  |
| It is difficult for me to reach for objects. |  |  |
| I have pain when walking. |  |  |
| My patience often runs out. |  |  |
| I feel that I am close to no one. |  |  |
| I am awake most of the night. |  |  |
| I feel that I am losing control. |  |  |
| I have pain when standing. |  |  |
| It is difficult for me to dress myself. |  |  |
| My energy quickly runs out. |  |  |
| It is difficult for me to stand for a long time (e.g., at the sink, at the bus stop). |  |  |
| I have constant pain. |  |  |
| I need a long time to fall asleep. |  |  |
| I feel that I am a burden to other people. |  |  |
| Worries keep me awake at night. |  |  |
| I feel that life is not worth living. |  |  |
| I sleep poorly at night. |  |  |
| It is difficult for me to get along with other people. |  |  |
| I need help when I want to move outside the house (e.g., a walking stick or someone who supports me). |  |  |
| I have pain when going up and down stairs. |  |  |
| I wake up feeling depressed. |  |  |
| I have pain when sitting. |  |  |
